# Kinome profiling reveals pathogenic variant specific protein signalling networks in MEN2 children with Medullary Thyroid Cancer

**DOI:** 10.1038/s41698-025-00919-4

**Published:** 2025-05-02

**Authors:** B. Rix, R. Chauhan, Z. Masoumi, E. Grönroos, C. E. Brain, O. K. Ogunbiyi, K. Swarbrick, C. Swanton, D. Bonnet, T. R. Kurzawinski, L. Izatt, N. Q. McDonald, W. Grey

**Affiliations:** 1 ProteoStem Lab, Centre for Blood Research, York Biomedical Research Institute, Department of Biology, University of York, York, UK; 2 Signalling and Structural Biology Laboratory, Francis Crick Institute, London, UK; 3 Cancer evolution and genome instability laboratory, Francis Crick Institute, London, UK; 4 Department of Endocrinology, Great Ormond Street Hospital, London, UK; 5 NIHR Great Ormond Street Hospital Biomedical Research Centre (BRC), London, UK; 6 Haematopoietic Stem Cell Laboratory, Francis Crick Institute, London, UK; 7 Clinical Genetics Service, Guy’s and St Thomas’ NHS Foundation Trust, London, UK; 8 Department of Medical and Molecular Genetics, School of Basic and Medical Biosciences, King’s College London, London, UK; 9 Institute of Structural and Molecular Biology, School of Natural Sciences, Birkbeck College, University of London, London, UK

**Keywords:** Proteomic analysis, Paediatric cancer

## Abstract

Multiple Endocrine Neoplasia Type 2 (MEN2) is an autosomal dominant disease caused by pathogenic variants in the receptor tyrosine kinase RET, with strong genotype-phenotype correlations. The development and progression of these tumours are not always predictable even within families with the same *RET* pathogenic variant, demonstrating a need for better understanding of the underlying molecular mechanisms. Precision molecular medicine is not widely used and the standard of care remains prophylactic thyroidectomy. This absence of curative approaches is exacerbated by the lack of novel therapeutic markers/targets. In this study, we investigated the functional kinome of 24 familial MEN2 patients. We identified MEN2 subtype and *RET* pathogenic variant-specific alterations in signalling pathways including mTOR, PKA, NF-κB and focal adhesions, which were validated in patient thyroid tissue. Overall, our study of MEN2 functional kinomes uncovers novel specific drivers of MEN2 disease and its pathogenic variant subtypes, identifying new potential therapeutic targets for MEN2.

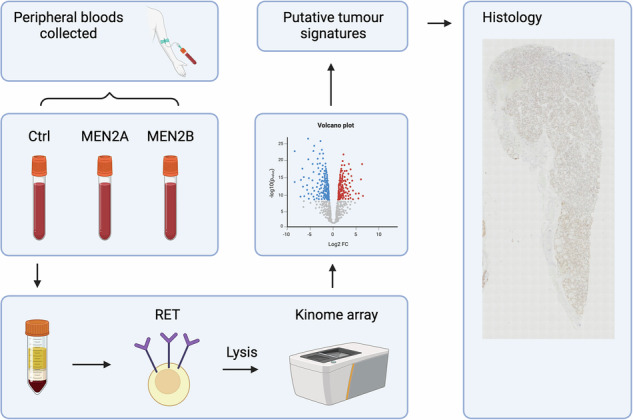

## Introduction

Multiple endocrine neoplasia (MEN) type 2 is an autosomal dominant condition which leads to the development of medullary thyroid cancer (MTC), adrenal phaeochromocytoma (PHAEO) and primary hyperparathyroidism (PHPT) caused by adenoma or hyperplasia^1^. MEN2 results from heterozygous constitutive activating pathogenic variants within the Rearranged During Transfection (*RET*) proto-oncogene, a transmembrane receptor tyrosine kinase^2^. Pathogenic variants show strong genotype-phenotype correlation and age-related penetrance^3^, allowing for treatment to be tailored to individual patients depending on the pathogenic variant^1,4–6^.

RET is responsible for the activation of numerous downstream signalling pathways governing cell proliferation and survival, playing an important role in embryogenesis, especially kidney morphogenesis, haematopoietic and germ cell differentiation, and the development of the enteric nervous system^2^. RET consists of 4 extracellular cadherin-like domains, an extracellular cysteine rich domain, a transmembrane domain, an intracellular juxtamembrane domain and an intracellular tyrosine kinase domain. Over 70 pathogenic variants in *RET* map along the length of the protein, including within the extracellular cysteine rich domains (e.g., C609, C611, C618, C620, C630, C634) and the intracellular tyrosine kinase domains (e.g., V804, A883, S891, M918)^7–9^. C609x-C634x, V804M and S891A pathogenic variants result in the MEN2 subtype MEN2A (95% cases^10^), while A883F and M918T lead to MEN2B.

Development of MTC is usually the first manifestation of MEN2. Due to the strong genotype-phenotype correlation between *RET* variants and MEN2-derived MTC, the risk of developing MTC can be assessed depending on the specific variant within *RET*^1^. According to the revised 2015 American Thyroid Association (ATA) guidelines, mutations at residues C634^1^ and A883^11^ are classed as high risk while the M918T mutation is classed as highest risk, in terms of developing MTC, with all other MEN2A variants classified as moderate risk^1^. MEN2A cysteine pathogenic variants constitutively activate RET through ligand-independent dimerisation^7,12^, whereas M918T, present in MEN2B patients, results in a conformational change that enables activation of both the dimer and monomer RET molecules, with RET activation and downstream signalling occurring even prior to exiting the endoplasmic reticulum^7,12,13^. Thus the rate of tumour development relies on the MEN2 subtype, with MEN2B accounting for only 5% of familial *RET* cases^10^, but presenting with the most aggressive form of the disease^1,7,8^.

Parafollicular C-cells progress through C-cell hyperplasia (CCH), non-invasive microcarcinomas followed by the development of MTC. The invasive disease state eventually leads to its spreading to local lymph nodes and later to the liver and bones^14,15^. Hereditary MTC (derived from hereditary MEN2 cases) is usually multifocal and bilateral^15^. In MEN2, CCH or MTC are seen to develop in >70% of MEN2A cases and 100% of MEN2B cases^10,16^, and are regularly present in MEN2 adolescents^17^.

Prophylactic thyroidectomy at an early age can prevent MTC^18^. The ATA recommends prophylactic thyroidectomy to be performed <1 year old for M918T patients and <5 years old for C634x and A883F patients^5^. Alternative studies have advocated for a potential ‘watch-and-wait’ approach for C634x carriers^19,20^, but age is still more significant than calcitonin levels^21^. For all other *RET* variants, a ‘watch-and-wait’ approach is advised, with surgery performed only with the onset of elevated serum calcitonin levels (a hormone produced by C-cells)^1^. However, not all children with MEN2 are identified through cascade genetic testing, as *de nov*o disease accounts for 90% of MEN2B and 7% of MEN2A cases. As such, the majority of the MEN2B cases are diagnosed at later stages, often already presenting with metastatic MTC, thus undergo surgery later than advised^22^, making thyroidectomy an ineffective treatment and non-curative^18,23^. Further to this, delayed treatment of MEN2B leaves 25% of the patients with very poor quality of life^18^, highlighting a need for the better understanding of specific underlying biological drivers of MEN2 to design novel treatment strategies.

While surgery is the only effective modality in treating MTC, some cases, especially those with metastatic disease, require additional systemic chemotherapy with multi tyrosine kinase inhibitors (TKIs) such as Vandetanib^24^. Yet over 20% of patients experience adverse side effects due to the broad kinase-targeting profile^24^. Children receiving Vandetanib often experience dose limiting diarrhoea, hypertension, QTc prolongation and demonstrate increasingly impaired sensitivity to levothyroxine, used to treat hypothyroidism, with elevated thyroid-stimulating hormone (TSH) levels^25–27^. Additionally, in vitro data suggests many multi-TKIs are not as effective against the so-called ‘gate-keeper’ pathogenic variants such as V804M^28^, with V804M acquisition being seen in clinic after unsuccessful multi-TKI treatments within an M918T MTC patient^29^. However, application of more selective RET kinase inhibitors, e.g., Selpercatinib, can stem tumour progression^29–31^, and have already shown to improve disease outcome for patients with MEN2-associated advanced MTC, with reduced toxicity, although early reports demonstrate resistance is acquired in some patients^31–37^. Development of such therapeutics, that are effective even after MTC onset or thyroidectomy, are imperative for increased patient survival and quality of life. However, RET-specific inhibitors do not always remain effective long term^34,36–39^ indicating the need for further molecular information and targets.

Without proper curative measures beyond thyroidectomy, improving the outcome for MEN2 patients relies on better understanding of the underlying disease mechanisms, to identify new biomarkers and design better therapeutics and treatment plans, particularly for the patients under watch-and-wait measures. Similarly, increased knowledge of the biochemical pathways affected by RET hyperactivity can give insight into potential novel therapeutic targets for MEN2 patients that haven’t undergone prophylactic thyroidectomy or have experienced disease relapse. As such, this study set out to identify novel biomarkers within inherited MEN2 patients, initially studying peripheral blood as a less invasive non-transformed cell type before validating identified signalling pathways in patient tumours. Due to the strong genotype-phenotype nature of MEN2, we particularly looked to identify pathogenic variant-specific drivers of the disease. We focused on studying the functional kinome of MEN2 patients to understand the downstream consequences of specific *RET* pathogenic variant activation and demonstrate variant-specific kinome remodelling and signalling pathway activity to gain new insights into the underlying biology, drivers of MEN2 and new putative therapeutic targets.

## Results

### Genotype-specific kinome alterations in MEN2

**Fig. 1:**
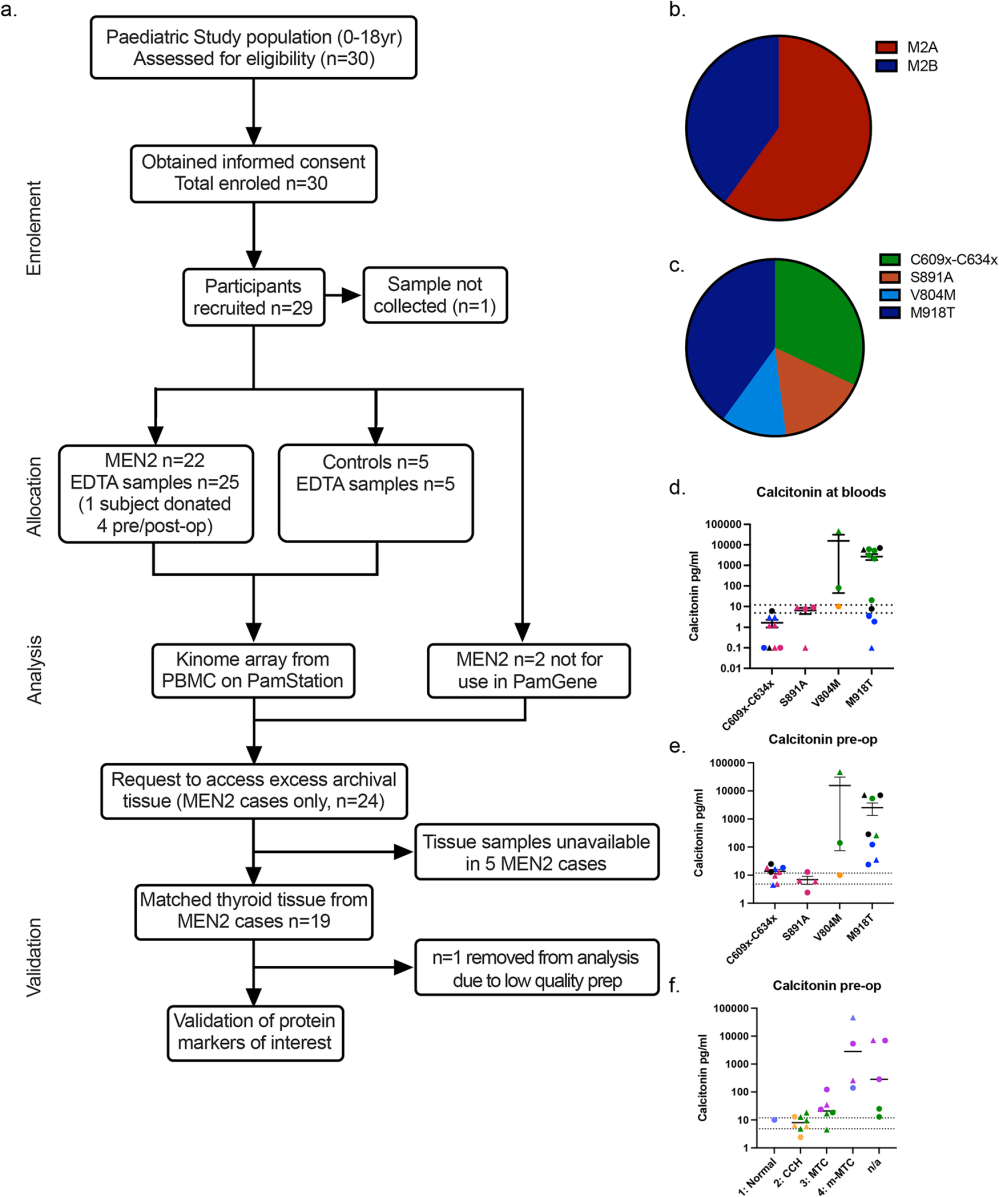
Patient cohort and experimental overview. **a** Consort plot of experimental pipeline and samples used at each step of the study. **b**,**c** Proportion of patient samples within the kinome array shown by (**b**) disease type and (**c**) pathogenic variant group. **d** Calcitonin levels within patients’ blood at the time taken for the kinome analysis. **e**, **f** Calcitonin levels within patients’ blood pre-thyroidectomy. **e** Data grouped by pathogenic variant and coloured by Histology group. Histology group 1 (Histologically normal) orange, group 2 (C-cell hyperplasia (CCH)) pink, group 3 (Medullary thyroid cancer (MTC)) blue, group 4 (Metastatic MTC (m-MTC)) green. Cases without thyroid tissue included in this study are shown in black. **f** Data grouped by Histology group and coloured by pathogenic variant (C609x-C634x green, S891A orange, V804M blue, M918T purple). Those without histology data are listed under n/a. **d**-**f** Error bars represent mean with standard error of the mean (SEM). The dotted lines at 11.8 pg/ml and 4.8 pg/ml, show when calcitonin levels are considered elevated above the healthy norm for male and female children respectively. Circles depict samples from males and triangles depict samples from females.

A total of 24 children with MEN2, carrying *RET* germline pathogenic variants (*n* = 16 MEN2A and *n* = 8 MEN2B patients) and 5 healthy controls were recruited into this study. This gave a total of 25 samples within the kinome assay, two samples were excluded due to poor sample preparation, and one MEN2B child donated samples on 4 separate visits alongside required clinical samples (Fig. 1a, Supplementary Fig. 1, Supplementary Table 2**)**. Peripheral blood was obtained at the point of recruitment for use in the kinome array (bloods for kinome). Excised thyroid samples from 19 of the MEN2 patients were donated for use in this study (1 was excluded from analysis due to low quality histological prep). For kinome analysis of peripheral blood mononuclear cells the MEN2 patients were further grouped by disease (*n* = 15 MEN2A, *n* = 10 MEN2B) **(**Fig. 1a, b. Supplementary Table 2**)**, pathogenic variant (*n* = 8 Cysteine Pathogenic variants, *n* = 4 S891A, *n* = 3 V804M, *n* = 10 M918T) **(**Fig. 1c. Supplementary Table 2**)** and the degree of MTC development where histology data was available (Group 1, *n* = 1 Patient tissue histologically normal. Group 2, *n* = 8 C-cell hyperplasia (CCH). Group 3, *n* = 6 Medullary thyroid cancer (MTC). Group 4, *n* = 6 Metastatic medullary thyroid cancer (m-MTC)). Two MEN2A cases with V804M presented with m-MTC, which is an unusually severe phenotype for MEN2 of moderate risk classification (Supplementary Table 2), where MTC development is generally later in life, presenting in a less severe form^1^.

In our MEN2 cohort, the calcitonin levels in blood samples collected for kinome array in patients with Cysteine pathogenic variants showed similar levels to that expected for healthy children ( < 11.8 pg/ml or <4.8 pg/ml, for males and females, respectively). At this time point for blood collection, 6 out of 10 M918T patients, 2 out of 3 V804M patients and 1 out of 4 S891A patients indicated disease progression with calcitonin levels above 11.8 pg/ml **(**Fig. 1d**)**. As thyroid tissue used in this study was not always obtained at the same time as the bloods used for the kinome analysis **(**Supplementary Table 2**)**, we also studied patient calcitonin levels pre-operation to investigate how patient blood calcitonin levels related to the observed thyroid histology (normal, C-cell hyperplasia, metastatic disease). In bloods taken pre-operation, high calcitonin levels were seen to be >11.8 pg/ml or >4.8 pg/ml (sex dependent) in 8 out of 9 Cysteine pathogenic variants carriers, 3 out of 4 S891A patients, 2 out of 3 V804M patients and all M918T patients **(**Fig. 1e, Supplementary Table 2**)**. The two V804M patients exhibiting elevated calcitonin levels, presented metastatic disease traits expected for MEN2B, not MEN2A. When analysed by MTC disease aggressiveness, patients presenting metastatic stage MTC showed elevated levels of calcitonin pre-op, corresponding with the invasive pathological presentation of MTC compared to the other 3 histology groups **(**Fig. 1f**)**.

To study the functional kinome of MEN2 patient cells carrying germline *RET* pathogenic variants, we isolated and lysed PBMCs and subjected the lysates to tyrosine and serine/threonine PamGene peptide-based microarrays to study functional kinase activity^40^.

**Fig. 2:**
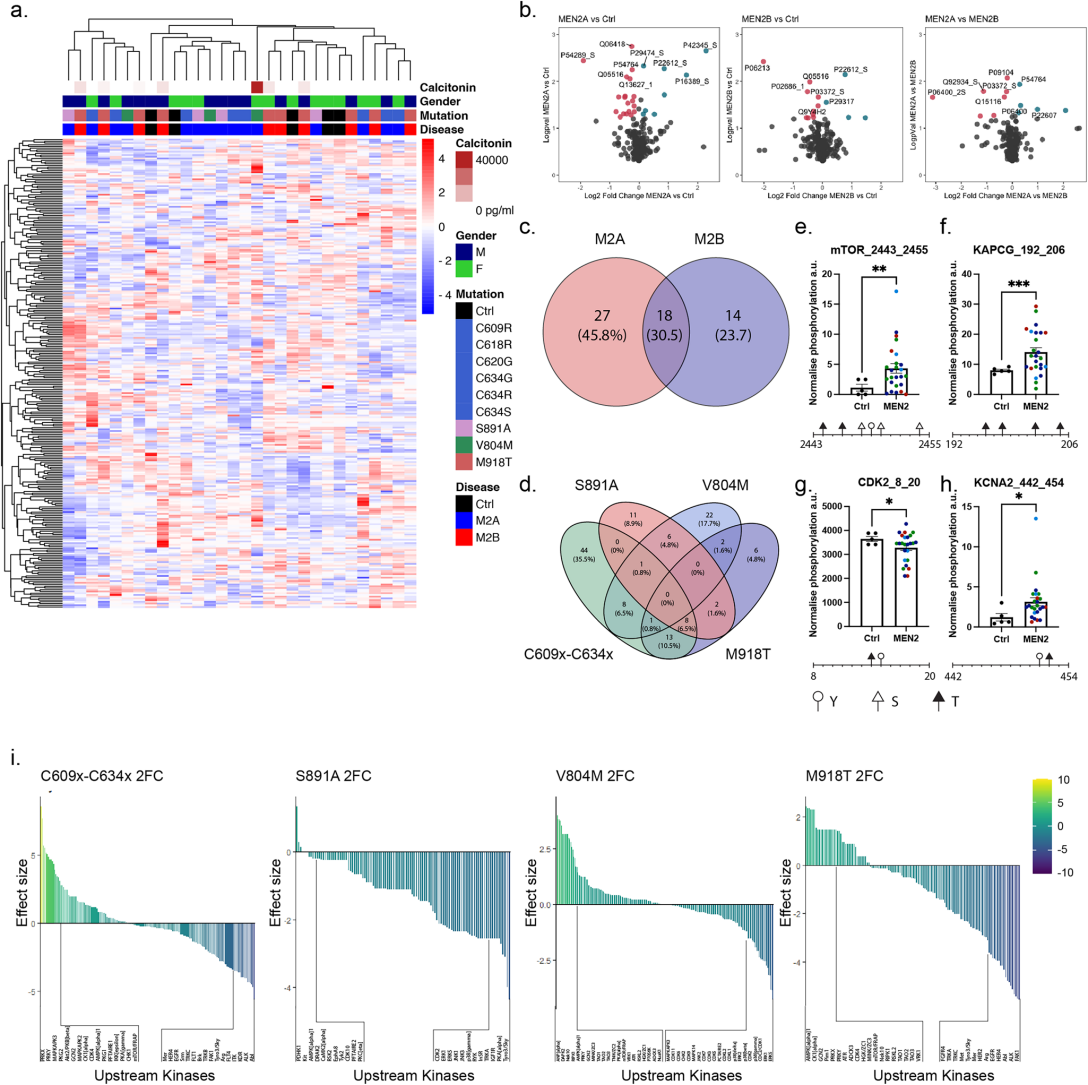
MEN2 patients’ functional kinome demonstrates pathogenic variant specificity. **a** Unsupervised heatmap of phospho-site targets identified within serine/threonine and tyrosine PamGene peptide arrays of MEN2 patient derived RET probed PBMCs. Signal intensities are represented as Z-scores calculated from the mean of all phosphorylation scores within the data set. **b** Volcano plots of phospho-sites with altered phosphorylation states when analysed between (left) MEN2A and controls (ctrls), (middle) MEN2B and ctrls, and (right) MEN2A against MEN2B. Significantly downregulated phospho-sites are shown in red. Significantly upregulated phospho-sites are shown in teal. *p* < 0.05. **c**, **d** Venn diagrams showing the number, percentage and overlap of significantly altered phospho-sites for each (**c**) disease and (**d**) pathogenic variant, all groups were compared to ctrls. **e**–**h** Phospho-sites with the greatest change in phosphorylation across all MEN2 patients are depicted against ctrls. Each point represents an individual patient. Colour represents pathogenic variant (C609x-C634x variants are shown in green, S891A in red, V804M in light blue and M918T in dark blue). * *p* < 0.05, ** *p* < 0.005, *** *p* < 0.0005, analysed by an unpaired t test with Welch’s correction. Locations of Tyrosine (Y, White circle), Serine (S, white triangle) and Threonine (T, black triangle) phosphorylation sites located within the phospho-peptides shown are depicted below each respective graph. **i** Charts show 2-fold change in differential activity for upstream kinases predicted upon PamGene results for each pathogenic variant. Kinases with the 10% highest and 10% lowest change in predicted activity are described below each plot.

Within our cohort, unsupervised clustering showed peptide phosphorylation varied between pathogenic variant/disease type, calcitonin levels and sex (Fig. 2a). Differential phosphorylation (MEN2 group vs. healthy controls) was observed at 27 phospho-sites specifically in MEN2A and 14 phospho-sites specifically in MEN2B, with a 30.5% overlap (18 phospho-sites) between MEN2A vs. controls and MEN2B vs. controls (Fig. 2b, c). When focussing on pathogenic variants, Cysteine pathogenic variants in extracellular domains showed the most homogeneous kinome alterations with more significantly different phospho-sites vs. controls compared to S891A, V804M and M918T (Fig. 2d, S2). When all MEN2 *RET* variants were grouped as a whole, the most significant differences were seen in the mammalian target of rapamycin (mTOR) between amino acids 2443 and 2455 (Fig. 2e), Protein Kinase CAMP-Activated Catalytic Subunit Gamma (KAPCG) amino acids 192 and 206 (Fig. 2f), Cyclin Dependent Kinase 2 (CDK2) between sites 8 and 20 **(**Fig. 2g**)**, and Potassium Voltage-Gated Channel Subfamily A Member 2 (KCNA2) between 442 and 454 **(**Fig. 2h**)**. The cumulative phospho-site changes between MEN2 sub-types and control were used to infer differential kinase activity (Fig. 2i). When C609-634x pathogenic variants are present Protein Kinase CAMP-Dependent X-Linked Catalytic Subunit (PRKX) and Protein Kinase Y-Linked (Pseudogene) **(**PRKY) show the highest increase in differential kinase activity, while Kinase Insert Domain Receptor (KDR), ALK Receptor Tyrosine Kinase (ALK) and ABL Proto-Oncogene, Non-Receptor Tyrosine Kinase (Abl) showed the greatest reduction. In S891A patients, Pyruvate Dehydrogenase Kinase 1 (PDHK1), KIT Proto-Oncogene, Receptor Tyrosine Kinase (Kit) and Protein Kinase AMP-Activated Catalytic Subunit Alpha 1 (AMPKɑ1) were the only kinases with an activity increase, all other proteins with a change in differential kinase activity were downregulated, with the largest reductions seen in Insulin Like Growth Factor 1 Receptor (IGF1R), Protein Kinase CAMP-Activated Catalytic Subunit Alpha (PKAɑ) and TYRO3 Protein Tyrosine Kinase (Tyro3/Sky). For V804M patients, the highest increase in differential kinase activity was seen in Atrial Natriuretic Peptide Alpha (ANPɑ), Death Associated Protein Kinase 2 (DAPK2) and NIMA Related Kinase 10 (Nek10), while the greatest reduction was seen in Cyclin Dependent Kinase (CDC2/CDK1), Mitogen-Activated Protein Kinase 3 (ERK1) and Mitogen-Activated Protein Kinase 7 (ERK5). In M918T patients, we again saw AMPKɑ1 as one of the highest increases in differential kinase activity, along with Casein Kinase 1 Alpha 1 **(**CK1ɑ) and Eukaryotic Translation Initiation Factor 2 Alpha Kinase 4 (GCN2). Similar to the C609-634x variants, Abl, ALK and also Protein Tyrosine Kinase 2 (FAK1) were seen to have the largest decrease.

Together, these data demonstrated that there were significant differences within the kinomes of the MEN2 patients compared to healthy controls, as well as between the kinomes of the MEN2 disease types. These differences in the kinomes were further shown to be specific to each pathogenic variant, with only partial overlap in the kinome changes seen between each pathogenic variant compared to healthy controls.

### p65 NF-κB signalling is increased in cysteine pathogenic variant carriers

**Fig. 3:**
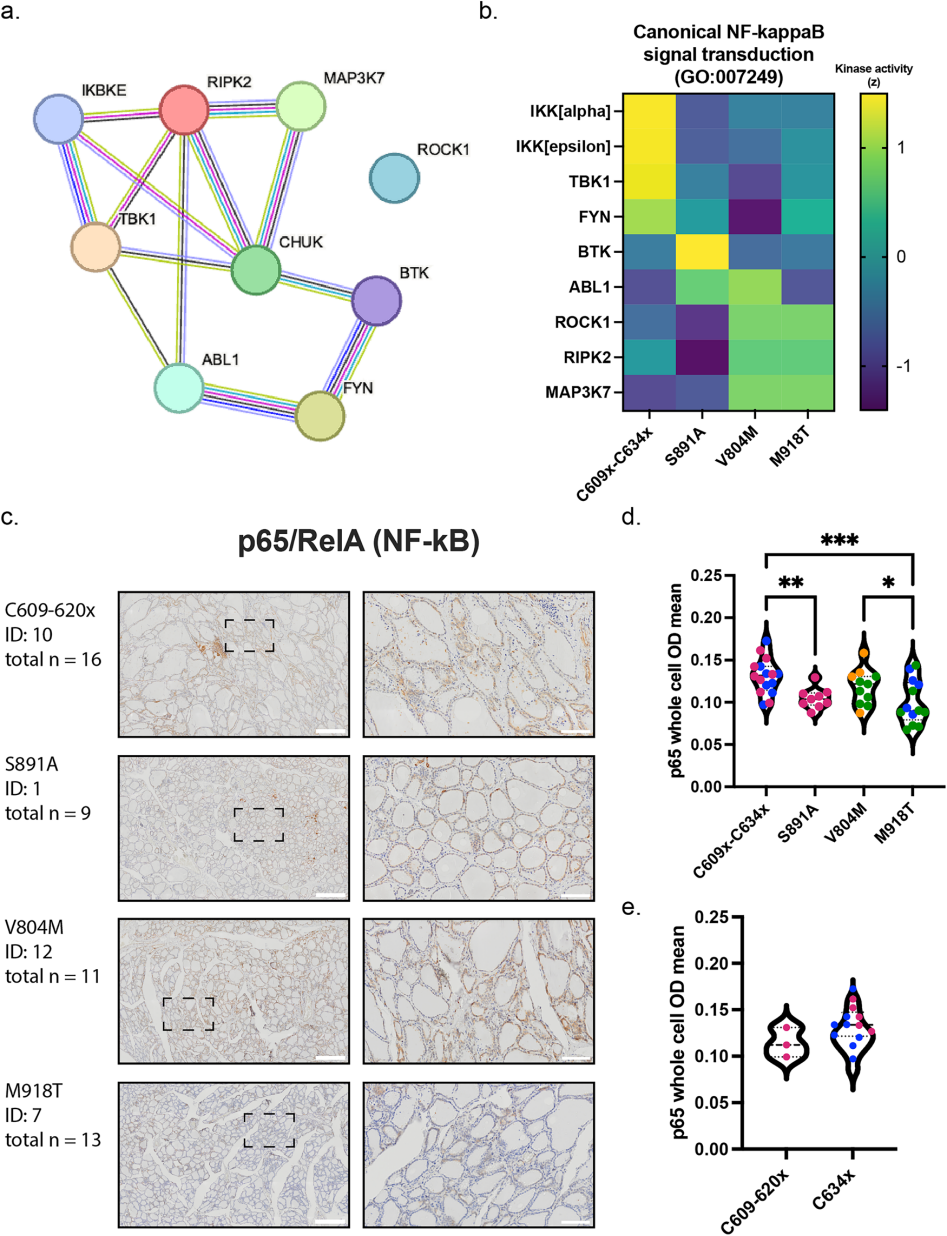
Disease progression within *RET* cysteine pathogenic variants is driven in part by NF-κB. **a** Network map of kinases within the NF-κB pathway (CHUK = IKKalpha. IKBKE = IKKepsilon). **b** Differential kinase activity of kinases shown in **a** Data shown is Z-scored. **c** p65 H-DAB immunohistochemistry of patient thyroid tissue. Labels show pathogenic variant, patient number and total number of samples for the pathogenic variant group. For images on the left, the scale bar shows 500 µm and the hashed line boxes show the location of images on the right. The scale bar for images on the right represents 100 µm. **d** Quantification of p65 cellular levels within thyroid tissue across all pathogenic variant groups. Colours show patient histology groups (group 1 (Histologically normal) orange, group 2 (C-cell hyperplasia) pink, group 3 (Medullary thyroid cancer) blue, group 4 (metastatic Medullary thyroid cancer) green). Analysed by One-way ANOVA followed by Brown-Forsythe test, * *p* < 0.05, ** *p* < 0.01, *** *p* < 0.001. **e** Data shown in **d** with Cysteine variants split by their metastatic potential C609-620X moderate, C634X high. Analysed by Mann-Whitney test.

We next sought to map the network of differential phospho-sites and kinase activity seen in MEN2A pathogenic variants. We focused on protein hits with differential phosphorylations altered in C609x-C634x patients, in particular their upstream kinases annotated to target those phospho-sites for STRING analysis, to thereby study connectivity and signalling pathway mapping with KEGG. We identified the Nuclear Factor kappa B (NF-κB) signalling pathway as enriched within Cysteine pathogenic variant patients (Fig. 3a, b). NF-κB pathway kinases Inhibitory-κB kinase ɑ (IKKɑ/CHUK), Inhibitor Of NF-κB Kinase Subunit Epsilon (IKKε) (canonical) and TANK Binding Kinase 1 (TBK1) (non-canonical) all increased in predicted kinase activity compared to controls in Cysteine Pathogenic variants. The remaining kinases; Bruton Tyrosine Kinase (BTK), ABL Proto-Oncogene 1, Non-Receptor Tyrosine Kinase (ABL1), Rho Associated Coiled-Coil Containing Protein Kinase 1 (ROCK1), Receptor Interacting Serine/Threonine Kinase 2 (RIPK2) and Mitogen-Activated Protein Kinase Kinase Kinase 7 (MAP3K7) showed increased activity in other pathogenic variants, especially V804M **(**Fig. 3b**)**. To validate the downstream mechanism, we analysed p65 protein within patient tumours (Fig. 3c, S3a). p65 (RELA proto-oncogene, NF-κB subunit) protein expression levels were significantly higher in Cysteine Pathogenic variant carriers than for S891A or M918T, and in V804M patients over M918T (Fig. 3d). This difference was greater within cell nuclei than the cytoplasm **(**Supplementary Fig. 3b**)**, with V804M nuclear p65 levels also being significantly higher (p65^hi^) compared to S891A **(**Supplementary Fig. 3b). When analysed by histology group, the most metastatic tumours (group 4) had the least p65 **(**Supplementary Fig. 3c**)**, significantly less than group 3 (p65^low^), but not groups 1 or 2. As C634x is classed in the 2nd highest metastatic category for *RET* pathogenic variants, while all other Cysteine pathogenic variants (C609-620x) are classed as moderate^1^, we further analysed p65 levels by C634x versus C609-620x. Separating Cysteine pathogenic variants, we found the mean cellular p65 level of C634x to be higher than C609-620x, however we could not state the relevance of this as we did not have sufficient samples for statistical analysis (Fig. 3e). Finally, Cysteine pathogenic variants and V804M both showed a significantly greater nuclear/cytoplasmic ratio of p65 than M918T (Supplementary Fig. 3b). The p65 nuclear/cytoplasmic ratio for V804M patient thyroid tissue was also significantly higher than that of S891A, indicating higher amounts of nuclear translocation within Cysteine pathogenic variants and V804M (Supplementary Fig. 3b). As such, our data suggest that MEN2 development for Cysteine pathogenic variant carriers within our study was, at least in part, through activation of the NF-κB signal transduction pathway.

### Alteration in focal adhesion complex component in MEN2B as a potential first step in metastasis

**Fig. 4:**
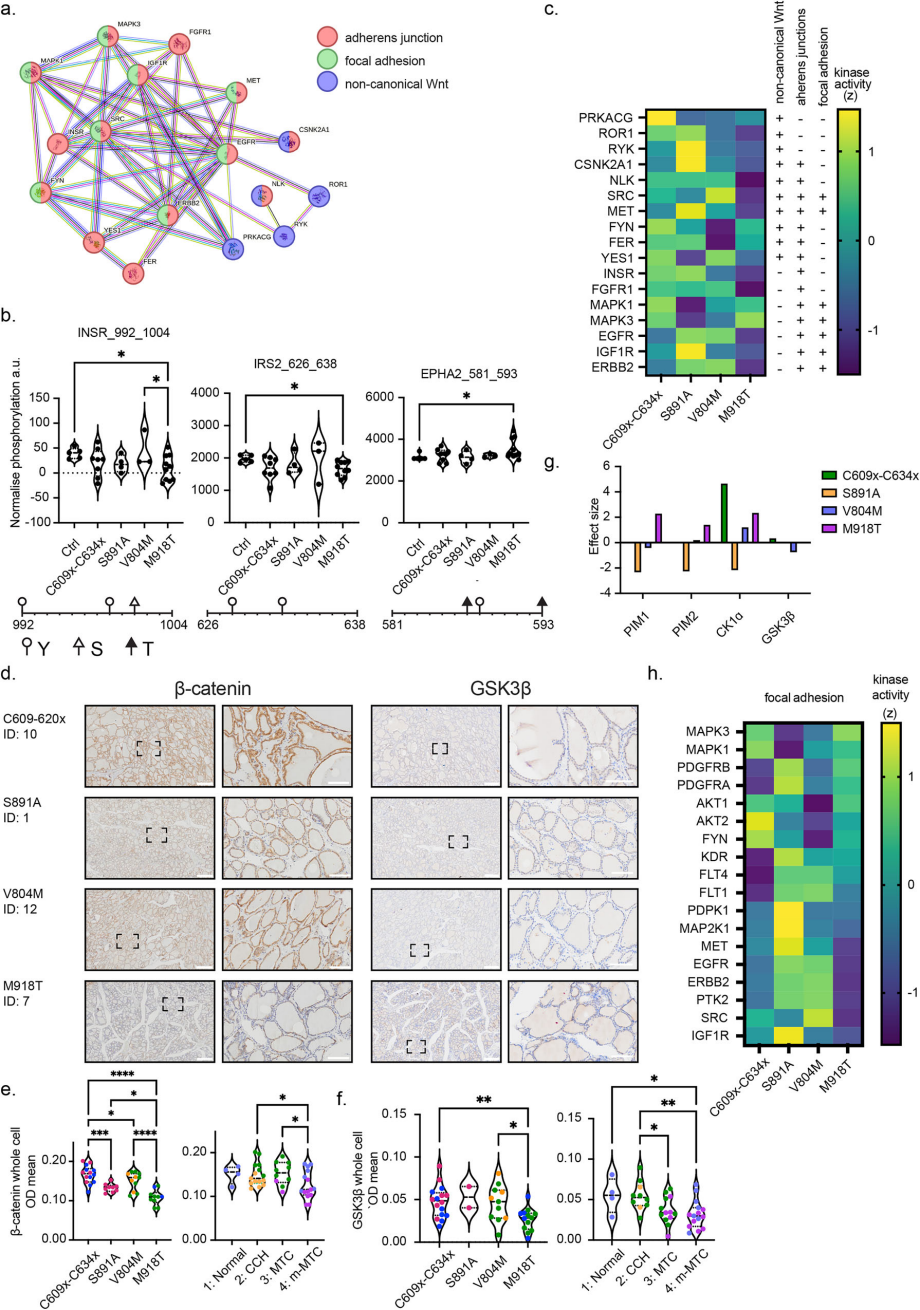
Focal adhesion signalling in pre-metastatic disease progression. **a** Altered kinase signatures for M918T carriers identified within the PamGene kinome array, along with their putative upstream kinases, were mapped to KEGG pathway networks comprising adherens junction (pink), focal adhesion (green) and non-canonical Wnt signalling (blue) modules. **b** Phosphorylation state of components of the focal adhesion pathway: Insulin receptor tyrosine kinase alpha subunit (INSR), Insulin receptor substrate 2 (IRS2) and Eph receptor tyrosine kinase A2 (EPHA2), identified by PamChip microarrays. INSR was analysed by One-way ANOVA followed by Brown-Forsythe test. IRS2 analysed by Kruskal-Wallis test. EPHA2 was analysed by Brown-Forsythe and Welch ANOVA. * *p* < 0.05. Phospho-sites located within each peptide are shown underneath (circles show threonine, white triangles show serine, black triangles show tyrosine residues. **c** Heatmap of the Z-scored differential activity of all kinases within the KEGG pathways shown in (**a**). **d** Immunohistochemistry H-DAB staining of patient thyroid tissue, probed for either β-catenin or GSK3β. The tissues are labelled with the pathogenic variant, patient participant number and the total sample number for that pathogenic variant. For each β-catenin and GSK3β, the scale bar for the images on the left represents 500 µm, with the hashed box showing the location of the closer in image on the right. For images on the right the scale bar represents 100 µm. Quantification of cellular **e** β-catenin and **f** GSK3β levels within patient tissues. Left, data is organised by each pathogenic variant, with colours representing the histology type (group 1 (Histologically normal) orange, group 2 (CCH) pink, group 3 (MTC) blue, group 4 (m-MTC) green). In the right graph data is organised by histology type, with colours representing the corresponding pathogenic variant (C609x-C634x green, S891A orange, V804M blue, M918T purple). Analysed by One-way ANOVA followed by Brown-Forsythe test, * *p* < 0.05, ** *p* < 0.01, *** *p* < 0.001, **** *p* < 0.0001. For β-catenin; C609x-C634x *n* = 14, S891A *n* = 9, V804M *n* = 11, M918T *n* = 10: Group 1 (histologically normal) *n* = 4, group 2 (CCH) *n* = 15, group 3 (MTC) *n* = 10, group 4 (m-MTC) *n* = 15). For GSK3β; C609x-C634x *n* = 16, S891A *n* = 2, V804M *n* = 11, M918T *n* = 13: Group 1 (histologically normal) *n* = 4, group 2 (CCH) *n* = 10, group 3 (MTC) *n* = 13, group 4 (m-MTC) *n* = 15. **g** The calculated change in kinetic effect of the PIM1, PIM2, CK1α and GSK3β kinases for each MEN2 pathogenic variant group. **h** Heatmap of the Z-scored differential activity of kinases within the focal adhesion pathway.

Given that p65^low^ appears not to be driving metastasis, we explored which signalling pathways are driving the most severe form of MEN2 disease by examining the most metastatic variant M918T. Performing network mapping, we identified altered kinases that mapped to three key candidate KEGG pathways, adherens junction, focal adhesion and non-canonical Wnt signalling **(**Fig. 4a**)**, with three phospho-sites linked to all three pathways (Fig. 4b). Components of the focal adhesion pathway, namely the Insulin receptor tyrosine kinase alpha subunit (INSR) and Insulin receptor substrate 2 (IRS2) had reduced phosphorylation states at amino acids 992-1004 and 626-638 respectively, in M918T PBMCs, compared with controls and V804M patients **(**Fig. 4b**)**. Another member of the focal adhesion pathway, Eph receptor tyrosine kinase A2 (EPHA2), showed an increased phosphorylation state in M918T variants between residues 581-593, compared to healthy controls **(**Fig. 4b**)**. Protein Kinase cAMP-Activated Catalytic Subunit Gamma (PRKACG), part of the non-canonical Wnt signalling pathway, showed higher phosphorylation with MEN2 PBMCs compared to controls, however this was not specific to MEN2B and was also observed in patients with S891A **(**Supplementary Fig. 4a**)**. The relative kinase activity of individual kinases in all three pathways was reduced in the majority of M918T cases (13 out of 17 kinases) (Fig. 4c), indicating a lack of non-canonical Wnt signalling and focal adhesion signalling.

Non-canonical Wnt signalling, focal adhesion and adherens junctions each share a common interacting protein, β*-*catenin, which has previously been reported to translocate to the nucleus in response to expression of an M918T-mimetic in mouse models and human metastatic MTC tissue^41^. To validate whether the pathways identified from the PamGene analysis are converging on β*-*catenin, and in fact influencing MEN2 disease progression, we obtained tumour samples from paediatric MEN2 patients to assess β*-*catenin protein levels and localisation. β-catenin cellular levels significantly decreased in line with increasing metastatic potential of the patient pathogenic variant **(**Fig. 4d, e**)**. This reduction was not specific to nuclear or cytoplasmic levels of β-catenin nor their β-catenin nuclear/cytoplasmic ratio, suggesting that classical nuclear functions may not be the main functional outcome **(**Supplementary Fig. 4b, c**)**. To investigate any correlation between the metastatic degree within a patient pathogenic variant group, we analysed M918T patients by MTC vs. metastatic MTC (m-MTC). We found a trend towards lower nuclear/cytoplasmic ratio of β-catenin within the m-MTC group **(**Supplementary Fig. 4d**)**. Only V804M patients had a significantly higher proportion (nuclear/cytoplasmic ratio) of β-catenin within tumour cell nuclei **(**Supplementary Fig. 4b**)**.

Cellular β*-*catenin levels are maintained via cytoplasmic degradation by glycogen synthase kinase-3 (GSK3β) in complex with APC Regulator of WNT signalling Pathway (APC) and Axin. The inhibition of this degradation, for example via the Wnt canonical pathway, leads to β-catenin stabilisation and nuclear translocation^42^. As such, we evaluated GSK3β activity and protein levels within patient kinomes and tissues respectively, to identify if GSK3β regulated the decrease in β-catenin observed in our patient groups. Within thyroid tissues, GSK3β protein levels significantly decreased within the whole cell **(**Fig. 4d, f**)** and cytoplasm **(**Supplementary Fig. 4e**)** of M918T variants. When analysed by extent of metastasis, MTC and m-MTC thyroid tissue was found to contain significantly less GSK3β than CCH tissue across the whole cell **(**Fig. 4d**)**, and less cytoplasmic GSK3β than both histologically normal thyroid tissue and CCH tissue **(**Supplementary Fig. 4e**)**. On the other hand, GSK3β protein levels did not differ between tissues from C609x-C634x, S891A or V804M variants. No change in the GSK3β activity was found within the kinome array for S891A or M918T variant groups. Only a marginal increase (0.34) was seen in C609x-C634x variants, opposed to a slight decrease (-0.76) in V804M variants (Fig. 4g).

In the absence of β-catenin nuclear translocation in M918T patients, we sought to explore the underlying mechanisms regulating the simultaneous decrease in β-catenin and GSK3β protein levels with variant pathogenic potential and metastasis level within tissue samples. In the PamGene kinome analysis, the serine/threonine kinases PIM1 proto-oncogene (PIM1), PIM2 proto-oncogene (PIM2) and CK1α altered in kinase effect in line with pathogenic variant potential (Fig. 4g), with CK1ɑ and PIM1 demonstrating the second and fourth greatest increase in kinase effect within M918T variants, respectively (Fig. 2i). In thyroid tissues PIM1 and PIM2 protein levels were significantly reduced within CCH, MTC and m-MTC tissues (Supplementary Fig. 6b, c). When analysed by pathogenic variants, PIM2 protein levels were significantly reduced in V804M and M918T variants compared to C609x-C634x variants (Supplementary Fig. 6c), while PIM1 protein levels did not significantly change between pathogenic variants (Supplementary Fig. 6b). Although we saw no increase in PIM1 protein levels between pathogenic variants, the simultaneous increase in PIM1 and CK1ɑ kinetic activity may be a disease driver within M918T.

We further studied the impact of all kinases within the focal adhesion pathway identified within our predictive analyses. We found a distinctive pattern in kinase activity for each patient pathogenic variant, with 50% of M918T kinases showing reduced activity compared to 28% reduced for S891A (Fig. 4h). However, the kinases’ phosphorylation status alone were in general unchanged from controls (Supplementary Fig. 4f). Together, our data indicates that MEN2 disease progression for M918T in our cohort may have been driven by the focal adhesion pathway allowing cell migration and metastasis. We found no evidence of changes in classical β*-*catenin nuclear activity in pre-metastatic tissue, but instead we observed a change in total β-catenin protein levels and propose β*-*catenin nuclear translocation occurs only during metastasis, while loss of protein levels at the cell surface is a pre-metastatic event.

## Discussion

It is well documented that MEN2 demonstrates a strong genotype-phenotype interaction, with a patient’s *RET* variant determining disease progression rates^3,5,6^. However, understanding of the biochemical and molecular drivers downstream of *RET* pathogenic variants in MEN2 is limited, with previous work focusing on RET downstream signalling without investigating pathogenic variant-specific contributions^43^ and with few molecular studies performed on primary patient material. Historically, studies have been limited by access to patient material and suitable methods to assess the impact of different disease variants. In this study, we explored the relationship between *RET* pathogenic variants and cellular kinome activity using PamGene kinome array technology to analyse the functional kinomes of 22 MEN2 paediatric patients (25 samples total, Supplementary Table 2) and 5 healthy controls, to identify overall MEN2 and *RET* pathogenic variant-specific alterations in downstream signalling in primary human cells.

Our results demonstrated that the functional kinome of MEN2 patients were predominantly dependent on the specific *RET* pathogenic variant, and that protein kinases such as mTOR, KAPCG, CDK2 and the potassium channel KCNA2 showed increased activation states across MEN2 pathogenic variants. Of these, only mTOR, known to promote cell proliferation, invasion and survival, has previously been linked to RET driven cancer^44,45^. Specifically, we identified significantly increased phosphorylation between mTOR amino acids 2443 and 2455 in MEN2 patients (Fig. 2e), covering an AKT Serine/Threonine Kinase (AKT) phosphorylation site and a mark of active mTOR previously identified as induced by activated RET^45^. The activation of the AKT/mTOR pathways occurs in >70% of MTC cases^45^ particularly in cases with germline *RET* pathogenic variants^44^. Within our cohort, Cysteine pathogenic variant carriers had AKT2 functional activity within the top 10% of increased effect size (Fig. 2i). Additionally, work from Tamburrino et al. indicates that mTOR activation occurs early in C-cell transformation and mTOR inhibition significantly reduces cell proliferation and motility^45^, which supports our results indicating a significant increase in mTOR activation within our patient cohort. Significant differences were also seen between KAPCG amino acids 192 and 206 (Fig. 2f). This region contains 4 sites of phosphorylation (T196, T198, T202, T205^46,47^) (Fig. 2f), with no previously reported link to RET. Other phospho-sites of significant difference included CDK2 between sites 8 and 20, which consists of T14 and Y15^46^ (Fig. 2g), dephosphorylation of these sites by Cell Division Cycle 25 (CDC25) is known to activate CDK2^48^. Finally, a significant increase in phosphorylation was seen upon the KCNA2 peptide between 442 and 454, within this region there are 2 phosphorylation sites in humans S451 and T452^49^ (Fig. 2h).

The NF-κB pathway is known to be essential for carcinogenesis within thyroid cancer^49–52^. In line with these previous studies, we found an increase in NF-κB signalling components, particularly within C609x-C634x variants (Fig. 3). For example, IkappaBalpha (IKBA) has been shown to have increased phosphorylation by IKKbeta kinase within *RET* C634x variant cells^49^ and NF-κB signalling within MTC can occur through p52, p65 and RelB^52^. However, within our data we found IKKalpha to have an increased differential kinase activity, not seen in the previous study to enhance the phosphorylation of IKBA^49^, while IKKbeta did not appear changed within our analysis (Fig. 3b). It should be noted that while we saw the kinase activity of NF-κB pathway members for V804M variants to be similar to that of M918T variants (Fig. 3b), this similarity may be the result of V804M variant cases studied being predominantly metastatic and not driven by the V804M variant alone. We also analysed the difference in NF-κB p65 within primary thyroid tissue, identifying higher nuclear levels of p65 (p65^hi^) compared to other MEN2 variants, as well as evidence for increased nuclear translocation (Supplementary Fig. 3b). Additionally, we analysed cellular p65 levels between Cysteine pathogenic variant groups based upon their degree of metastasis but found no strong evidence to support this (Fig. 3e). Together this demonstrates that in primary patient tumour material NF-κB signalling is more predominant in cysteine-rich variants (p65^hi^) as opposed to the most aggressive M918T variant (p65^low^), arguing for the need to study primary patient material in addition to other model systems.

Kinome activity from MEN2B patients with the M918T pathogenic variant identified that the adherens junction, focal adhesion and the non-canonical Wnt signalling pathway were key impacted processes within our MEN2B cohort **(**Fig. 4**)**, particularly observed was a decrease in the activity of focal adhesion kinases (Fig. 4h). INSR, IRS2 and EPHA2, all protein members of these pathways, showed significant changes in phosphorylation status of the phospho-peptides included in the kinome array (Fig. 4b). INSR 992-1004 contains 3 phosphorylation sites, Y992, Y999 and S1001 (Fig. 4b). Phosphorylation of Y992 leads to enzymatic inhibition whereas phosphorylation of Y999 leads to enzymatic activation^53,54^. There is currently no information regarding the resulting activity or downstream impact of S1001 phosphorylation. The arrayed IRS2 626-638 peptide contains Y628 and Y632 (Fig. 4b), with unphosphorylated Y628 promoting IRS2 binding with the insulin receptor^55^. Eph receptor A2 (EPHA2; also part of the focal adhesion pathway) phosphorylation levels at phospho-sites 581–593 were upregulated in M918T (Fig. 4b). The 581-593 peptide of EPHA2 contains T587, Y588 and T593 (Fig. 4b), and autophosphorylation of Y588 promotes enzymatic activity and downstream signalling^56^. EPHA2 is reported to promote cell migration within thyroid cells both via and independently of FAK^57^, and also to mediate thyroid cell invasion through pAKT^58^. Together, the phosphorylation events identified with the kinome array implied the action of invasive adhesion functions within MEN2B metastasis.

In addition, our data suggested a link to Wnt signalling. This pathway can be classified into the canonical pathway, involved in cell proliferation/plasticity, and the non-canonical pathway, involved in cell migration^59^. Direct RET phosphorylation of β-catenin has previously been shown to support M918T tumour progression in immortalised cells, overexpressed mouse models and metastatic MTC in human thyroid, promoting β-catenin nuclear translocation and the transcription of genes similar to that when acting through the Wnt canonical pathway^41^. Within our study, we saw no difference in the Nuclear/Cytoplasmic ratio of β-catenin between Cysteine pathogenic variants, S891A and M918T (Supplementary Fig. 4b). As such we propose that neither direct RET phosphorylation of β-catenin, nor the canonical Wnt β-catenin role, may be influencing the difference in disease metastasis seen between MEN2A and MEN2B patients in our patient cohort. However, similar to our findings, Gujral et al. showed that β-catenin within human primary MTC was predominantly membranous and post metastasis β-catenin became 71% nuclear^41^. In our cohort, we found overall β-catenin levels within cells (Fig. 4 + S4) to significantly decrease correlating with pathogenic variants’ effecting metastatic potential. This is opposed to Gujrals’ findings in cell culture that β-catenin shRNA resulted in reduced cell invasion and tumour size^41^. This may reflect that fast-growing cell line models are not directly relevant for the MEN2 disease system within human tissue. β-catenin loss is known to associate with metastasis and poor prognosis of numerous cancers, such as colorectal cancer^60^, breast cancer^61^ and non-small-cell lung cancer^62^. Loss of β-catenin, particularly at the membrane, is proposed to aid Epithelial to Mesenchymal Transition (EMT) through reduction of its cell-adhesive properties, leading to cell migration and metastasis^60,61,63^. EMT transition, in addition to the reduction in focal adhesion kinases that we observed in the blood of more metastatic patients (Fig. 4h), suggests a mechanism through which cells metastasise within MEN2B patients. As such, we propose β-catenin may act through the focal adhesion pathway and non-canonical Wnt signalling pathway in pre-metastatic tissue as seen in our study, promoting loss of cell adhesion and progression of EMT, then switching to a post-metastatic state to act through both or individually the canonical Wnt pathway to promote oncogenic gene transcription and metastasis. Overall, our immunohistochemistry and kinome pathway analyses are unequivocal in supporting a non-canonical role for β-catenin promoting cell metastasis through non-canonical Wnt signalling.

We next aimed to better understand the upstream mechanism underlying β*-*catenin levels degradation and reduction in line with the MEN2 pathogenic variant, to identify clinically actionable targets for the future. Despite a decrease in GSK3β protein levels in line with the MEN2 pathogenic variant and degree of metastasis (Fig. 4f, S4e), we found its activity to remain the same across groups whilst PIM1, PIM2 and Ck1ɑ all showed an increase in kinase activity (Fig. 2i). PIM1 is known to phosphorylate GSK3β at Serine 9^64^. Serine 9 is located within the N-terminal tail of GSK3β and its phosphorylation inhibits GSK3β activation by blocking the substrate binding site^65^ and targets GSK3β for degradation^66^. CK1ɑ however phosphorylates and primes β*-*catenin for phosphorylation and degradation by GSK3β^67^. Thus, an increase in CK1ɑ activity could facilitate priming of β*-*catenin for degradation by the active GSK3β, not yet phosphorylated by PIM1. As such, PIM1 would improve cell survival as well as metastasis by reducing cell adhesion via β-catenin despite its inhibitory impact on GSK3β. Further investigation into this pathway and potential role in regulating metastasis in MEN2B patients is required to confirm whether PIM1 could be an actionable clinical target.

The scope of this study has focused on the functional kinome within MEN2 subtypes, covering 22 MEN2 paediatric patient PBMCs with validation in 18 primary tumour biopsies. Although we aimed to be as broad as possible, this study was limited in patient age and sex based upon sample collection at GOSH and GSTT. Furthermore, not all patients had elevated calcitonin levels at the time of sampling for the kinome assay, as some had previously raised levels and undergone successful thyroid surgery prior to the blood collection. Those that had high calcitonin were the V804M and M918T cases with metastatic medullary thyroid cancer (histology Group 4), with two of three V804M patients presenting metastatic traits more expected for M918T. Having obtained the PBMCs predominantly before disease onset (without elevated calcitonin levels), our study cannot describe probable changes in the kinomes after disease onset. However, this does enable our findings to give more insight into pre-onset markers and potential targets, rather than targeting post-disease onset. Finally, validation of our findings within tumour tissue was only between patient groups, as no healthy thyroid tissue was available for age-matched controls. As such, we cannot state if the β-catenin, p65 or PIM kinases cellular levels shown within this study were up or downregulated compared to healthy thyroids, only between pathogenic variants and histology types.

Overall, the strong genotype-phenotype interaction seen here within MEN2 patient kinomes points to pathogenic variant-specific drivers of MEN2 disease. Our data provides further understanding of the molecular mechanisms underlying specific MEN2 subtypes and this work combined with others aims to identify novel biomarkers and molecular targets for use with patient treatment plans, as well as aiding those with advanced disease for whom prophylactic surgery is not an option and new treatments are needed.

Our work contrasts with some previous studies of the biochemical pathways underlying RET metastasis, however this is likely due to the different tissues and model systems used and underlines the need to cross-validate findings in primary patient material^43^. This further demonstrates the need for new insights and understanding of MEN2 disease drivers, in particular by patient subtypes, as we have analysed here. As better laboratory-based models are developed, potential biomarkers of MEN2 identified within this study would be interesting targets to validate. Overall, the increased availability of biomarkers enabling targeted and personalised treatment for MEN2 patients is providing the possibility of preventative healthcare, preventing MTC with minimal treatment side effects^68–70^.

## Methods

### Patient information

Patient inclusion criteria entailed a MEN2 diagnosis, and a confirmed heterozygous germline *RET* pathogenic variant. The patients were children under the care of clinical genetics and paediatric endocrine teams at two tertiary London hospitals, Great Ormond Street Hospital and Guy’s and St Thomas’ NHS Foundation Trust. 24 MEN2 paediatric cases, aged 1-16, enrolled in the study between 2017 and 2019. At the time of enrolment, some of the MEN2 cases had already undergone thyroid surgery, others had surgery at enrolment and some cases had prophylactic thyroid surgery after enrolment. The timing of surgery was determined by their clinical teams, based on the disease presentation, biochemical tests, imaging results and known genotype. At the time of enrolment, no cases had started on Tyrosine kinase inhibitors (TKI), but 6 cases subsequently commenced the treatment^34^. Patient demographics, genetic results, biochemical tests, and tumour characteristics were extracted from electronic hospital records. Calcitonin levels were measured immediately prior to operation and at the time of the kinome array. In addition, 5 healthy paediatric controls, aged 12-18, were recruited in 2019.

### Ethical Approval

The study was approved by the NRES Committee South Central- Hampshire A (REC 13/SC/0574), this research was performed in accordance with the Declaration of Helsinki. The local ethics and institutional reviews boards were ‘Guy’s and St Thomas NHS Foundation Trust, London, co-sponsored with King’s College London Local research ID RJ/114/N093 IRAS 118008’, ‘Great Ormond Street Hospital, London Local research ID 18BB02’ and ‘King’s College Hospital, London Local research ID KCH14-124’. All human samples, associated clinical data and tissue samples were obtained after informed written consent.

### PamGene Kinome Array

Peripheral blood was collected in EDTA tubes (BD biosciences) after informed consent at the point of recruitment at Great Ormond Street Hospital or Guy’s and St Thomas’ Hospital (London). At this timepoint for blood collection, some patients had already undergone total thyroidectomy (Supplementary Table 2). Peripheral blood mononuclear cells (PBMCs) were isolated by density centrifugation using Ficoll-Paque (GE healthcare). The PBMCs were thoroughly washed in PBS and lysed in MPER buffer with 2x Halt Protease and Phosphatase Inhibitors (Thermo Scientific) on ice for 30 min. The lysates were centrifuged at >12,000 g for 10 mins at 4 °C, and the supernatants were retained for protein quantification using a BCA kit (Pierce) immediately followed by kinome analysis via PamChip microarrays. The microarrays consisted of specific immobilised peptides that can be phosphorylated by the active kinases present in the lysates. These phosphorylation events were then measured and compared at the peptide level. PamChip microarrays were run using 2 µg (Protein tyrosine kinase assay (PTK)) or 0.25 µg ((Serine Threonine kinase assay (STK)) of protein as per PamGene assay protocols (PamGene) using a Pamstation12 instrument. The results were initially analysed using the Bionavigator software (PamGene), subject to kinetic quality control and VSN normalisation. Data was plotted using Rstudio, with peptides containing tyrosine phosphorylation sites denoted simply as ‘peptideID’ and peptides containing serine or threonine phosphorylation sites denoted as ‘peptideID_S’.

### Kinome pathway analyses

Gene ontology analysis was performed using String-db.org and upstream kinases of phospho-sites identified within the PamChip microarrays were predicted on PamGene analysis pipeline assignment. Overall effect size on the kinome for each upstream kinase was based on the mean log2 fold change of the PamGene targets affected by that kinase. All identified upstream kinases and PamGene targets were then pooled and analysed on String-db.org and KEGG as previously described^40,71,72^.

### Tumour histology

Primary patient embedded thyroid tissue biopsies obtained at thyroidectomy from MEN2 cases were procured at Great Ormond Street Hospital or Guy’s and St Thomas’ Hospital (London). Each sample was then processed by The Francis Crick Experimental Histology team. The thyroid tissues were formalin fixed, paraffin-embedded, blocked for peroxidases with 1.6% H_2_O_2_ in PBS for 10 mins, and then blocked in 1% BSA in PBS. Immunohistochemistry (IHC) staining was performed either manually (β-catenin and NF-κB p65) or using the Leica Bond Platform (Leica Biosystems) (GSK3β, PIM1, PIM2). DAB was used for detection of the protein of interest, haematoxylin was used for cell nuclei detection. Full details of all the antibodies used are provided in Supplementary Table 1. Each slide, containing 1to 7 thyroid tissue foci per patient, was imaged on either an Olympus VS120 Slide Scanner (β-catenin, p65) or Olympus VS200 Slide Scanner (PIM1, PIM2, GSK3β), at 20x magnification. For analysis each separate section of tissue was treated independently.

### QuPath Histology analyses

The images were acquired on the microscope as .vsi files and were analysed using either QuPath v0.3.2 (β-catenin, p65) or QuPath v0.5.1 (PIM1, PIM2, GSK3β)^73^. A subset of images was used as a training dataset to produce a Random trees-based pixel classifier which could identify tissue within an image. Each individual piece of tissue was manually outlined using the *Polygon tool*, followed by finer annotation of the tissue using the in-house defined Random trees pixel classifier to obtain one annotation per tissue piece covering the full tissue section. Any thyroid tissue regions missed by the pixel classifier were then added into the annotation manually. Cell detection was performed using QuPath’s cell segmentation algorithm. The full script is provided within the file Supplementary Information and is available at www.proteostem.co.uk. The mean DAB protein staining per cell region (whole cell, nuclei, cytoplasm, nuclear/cytoplasmic) was exported per tumour for data organisation in Rstudio v4.2.3^35^.

### Statistical analysis

All statistical tests were performed on either GraphPad (version 10.1.1, GraphPad Software, Boston, Massachusetts USA, www.graphpad.com) or PamGene software^74^. All IHC protein DAB intensities were analysed between patient pathogenic variant and histology groups by One-way ANOVA followed by Brown-Forsythe test in GraphPad, *p* value < 0.05 was considered as statistically significant. All other statistical tests and p values are noted in the figure legends.

## Supplementary information

Supplementary Information

Supplementary Table2

## Data Availability

All data is indexed in the Francis Crick Institute file store and is available upon reasonable request.
