# Supplementary material for: Kinome profiling reveals pathogenic variant specific protein signalling networks in MEN2 children with Medullary Thyroid Cancer
Source: NPJ Precis Oncol. 2025 May 2;9:125. doi: 10.1038/s41698-025-00919-4 (PMC12048619; doi:10.1038/s41698-025-00919-4)
Supplement: Supplementary file 1 — Supplementary Information [file 41698_2025_919_MOESM1_ESM.pdf]

Supplementary Information

a.

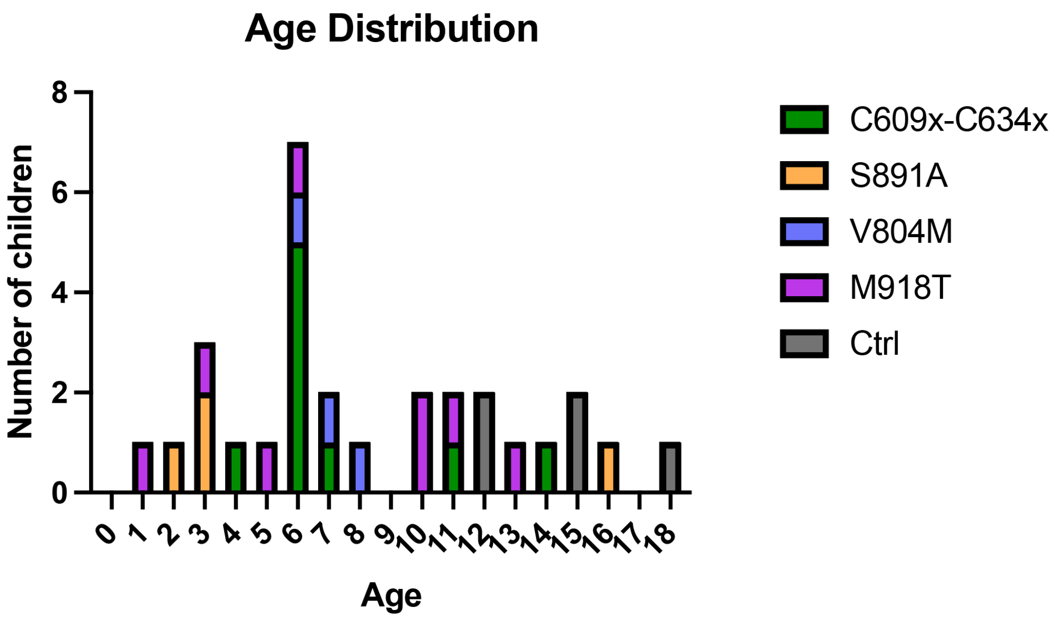

b.

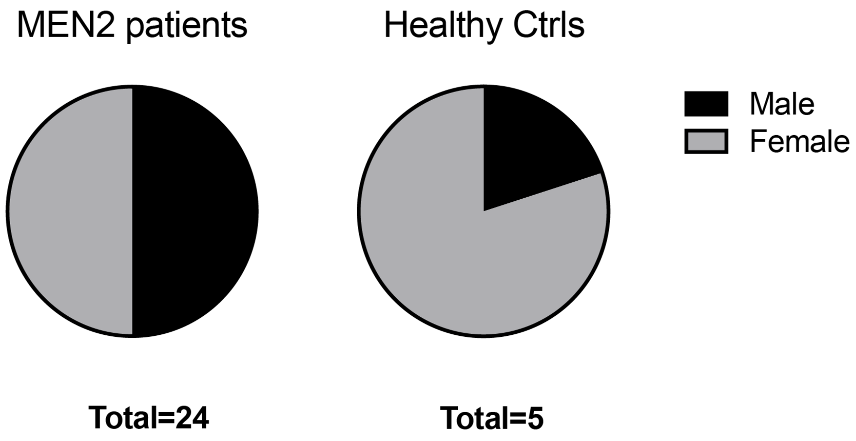

Supplementary Figure 1. **Patient cohort.** a) Patient ages and distribution. Colours show patients' *RET* pathogenic variant. b) Proportion of MEN2 patients and healthy controls by Sex. Black male, grey female.

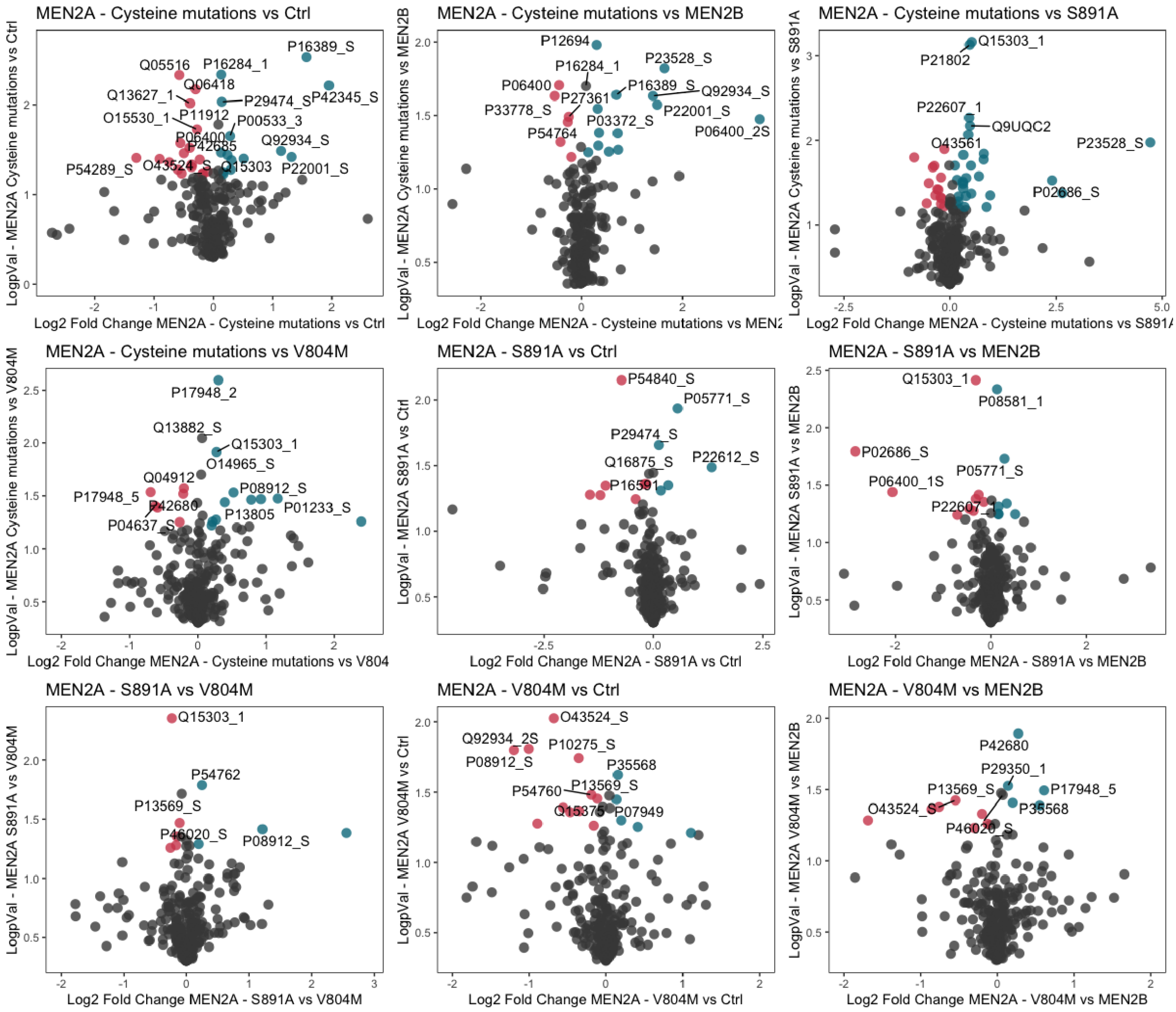

Supplementary Figure 2. **MEN2 change in phospho-sites shows pathogenic variant specificity.**

Phospho-site levels for each pathogenic variant group identified through PamChip microarrays were compared against controls and other pathogenic variant groups. Significantly downregulated sites between groups are shown in red, whilst significantly upregulated sites shown in teal.

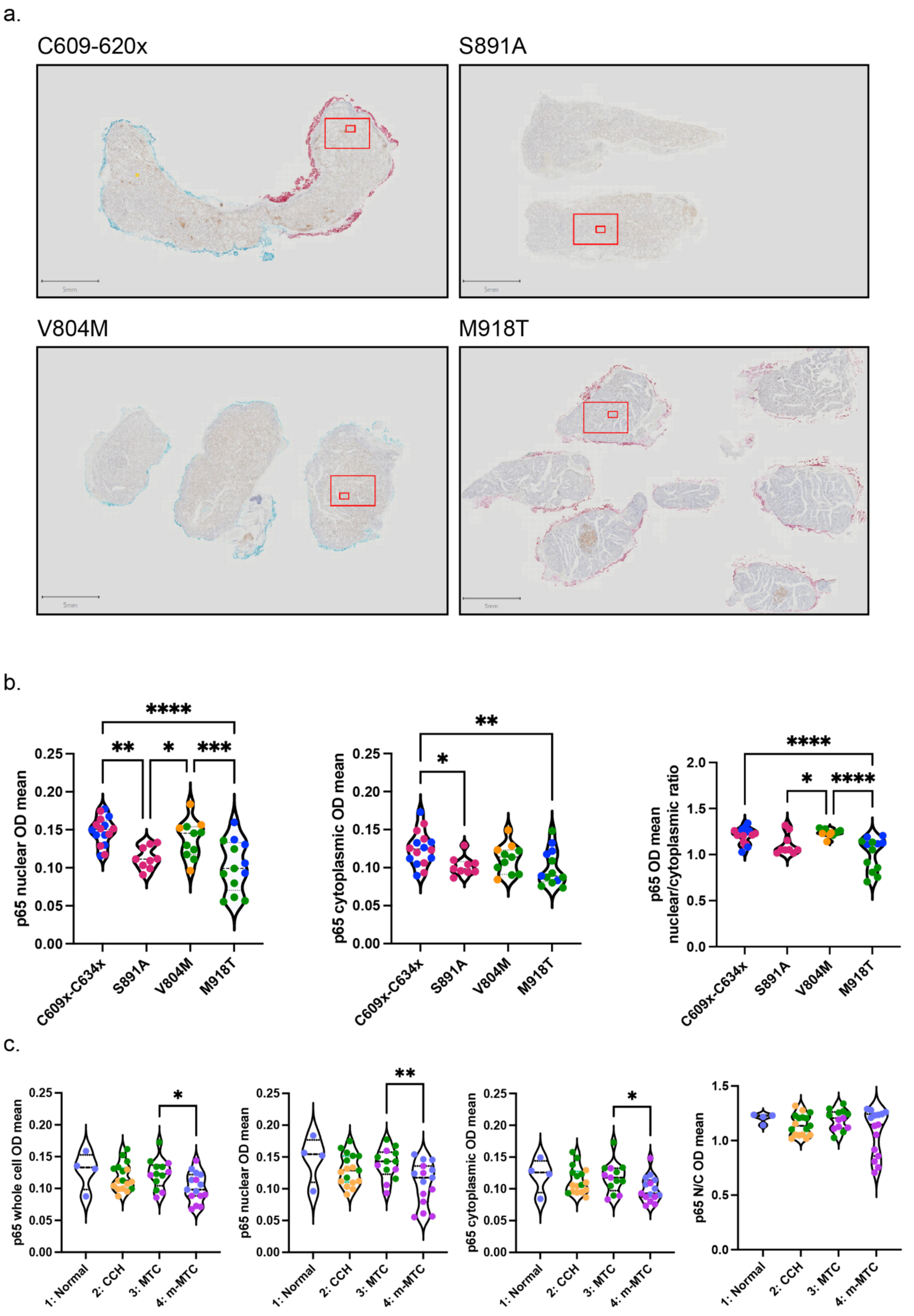

Supplementary Figure 3. p65 protein levels are significantly higher within *RET* C609x-C634x variants. a) Representative images of patients excised thyroid tissue, stained for p65,

with scale bar showing 5 mm. Red boxes show regions shown in Figure 4c. b) Quantification of p65 protein levels within cell (left) nuclei, (middle) cytoplasm, and the (right) nuclear / cytoplasmic ratio, shown by pathogenic variant group. Colours show each tissues histology type (group 1 (Histologically normal) orange, group 2 (CCH) pink, group 3 (MTC) blue, group 4 (m-MTC) green). c) p65 levels split by histology group. Colour represents patients' pathogenic variant (C609x-C634x green, S891A orange, V804M blue, M918T purple). b-c) Analysed by One-way ANOVA followed by Brown-Forsythe test, * $p < 0.05$, ** $p < 0.01$, *** $p < 0.001$, **** $p < 0.0001$. C609x-C634x $n = 16$, S891A $n = 9$, V804M $n = 11$, M918T $n = 13$: Group 1 (histologically normal) $n = 4$, group 2 (CCH) $n = 17$, group 3 (MTC) $n = 13$, group 4 (m-MTC) $n = 15$.

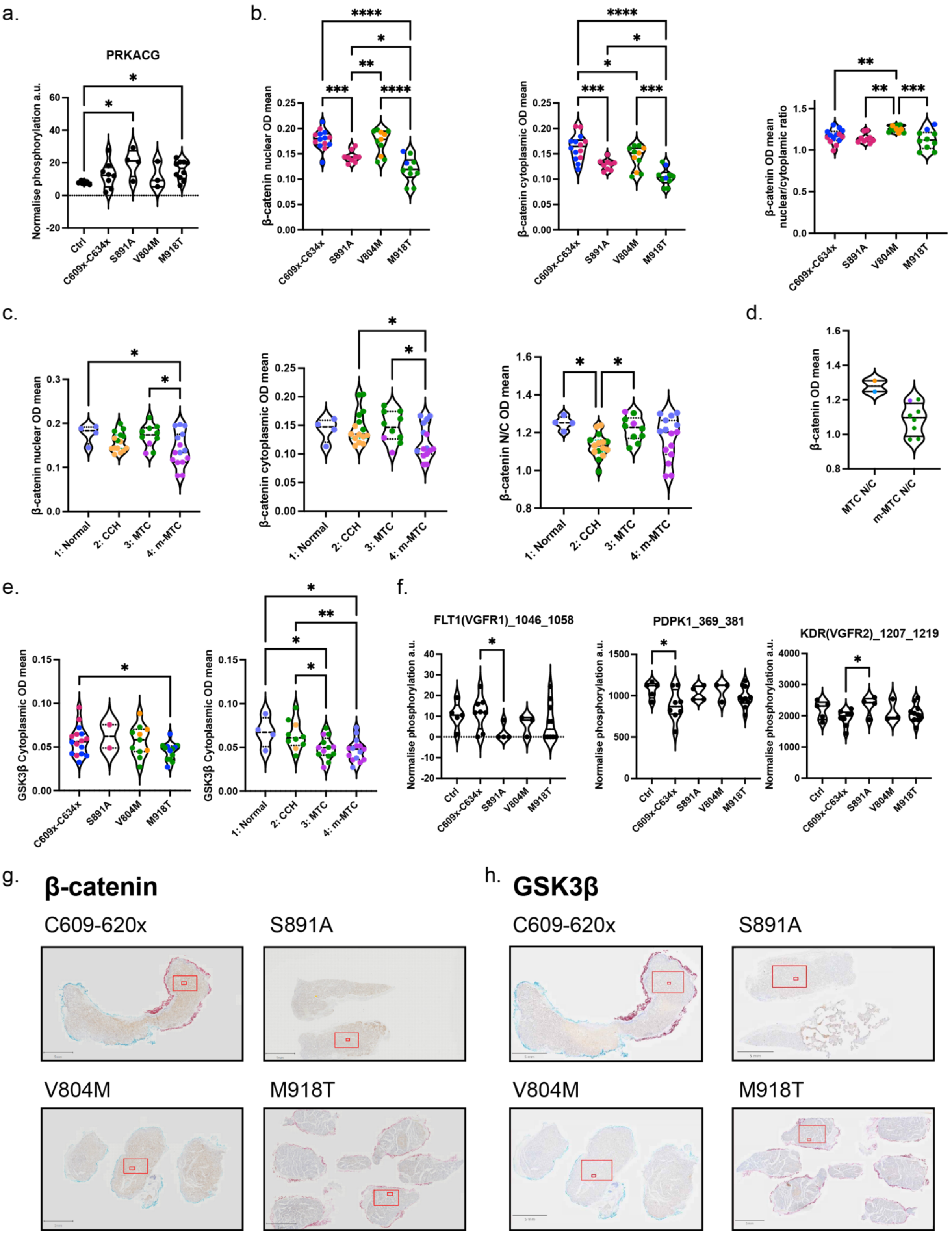

Supplementary Figure 4. β-catenin and GSK3β protein levels decrease in line with pathogenic variants' metastatic potential.

a) Normalised phosphorylation level of PRKACG for each group. b) Quantification of β-catenin levels within patient tissues. (Left) nuclear, (middle) cytoplasmic, (right) nuclear / cytoplasmic ratio. Colours represent the patients histology type (group 1 (Histologically

normal) orange, group 2 (CCH) pink, group 3 (MTC) blue, group 4 (m-MTC) green). c) Quantification of β-catenin levels within patient tumours split by histology group. Colour represents patients *RET* pathogenic variant (C609x-C634x green, S891A orange, V804M blue, M918T purple). d) β-catenin levels within tissue cells of M918T patients with either MTC or m-MTC. Colours show individual patients. e) Cytoplasmic protein levels of GSK3β within tissue samples. Data is organised either by patients' pathogenic variant or histology type, graphs are coloured in the same manner as panels b and c, respectively. f) Phosphorylation state of kinases (left) Fms related receptor tyrosine kinase 1 (FLT1), (middle) Phosphoinositide-dependent Kinase-1 (PDPK1) and (right) Kinase insert domain receptor (KDR). g) Thyroid tissue from 1 patient of each *RET* pathogenic variant group. Red boxes show tissue regions shown within Figure 3d. Scale bar show 5 mm. a-c and e-f were analysed by One-way ANOVA followed by Brown-Forsythe test, * $p < 0.05$, ** $p < 0.01$, *** $p < 0.001$, **** $p < 0.0001$.

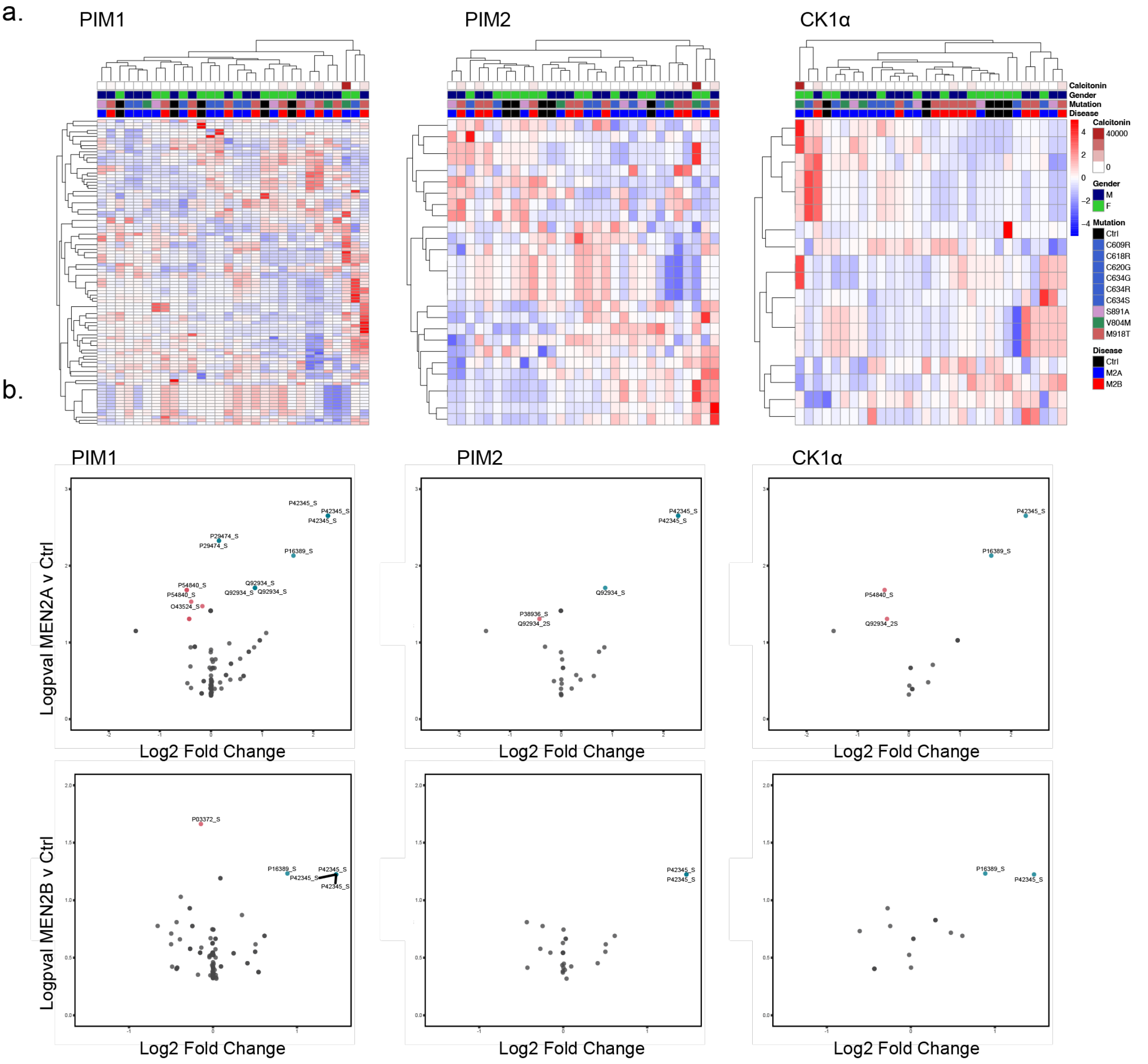

Supplementary Figure 5. PIM1, PIM2 and CK1a kinase activity.

a) Unsupervised heatmaps of PIM1, PIM2 or CK1α phospho-site targets, which were identified within the serine/threonine PamGene array of RET probed mononucleocytes. The signal intensities are represented as Z-scores as explained in Figure 2. b) Volcano plots of the phospho-sites with altered phosphorylation states when analysed between (top) MEN2A and controls (ctrls) and (bottom) MEN2B and ctrls. Phospho-sites that were significantly up or downregulated are shown in teal or red, respectively.

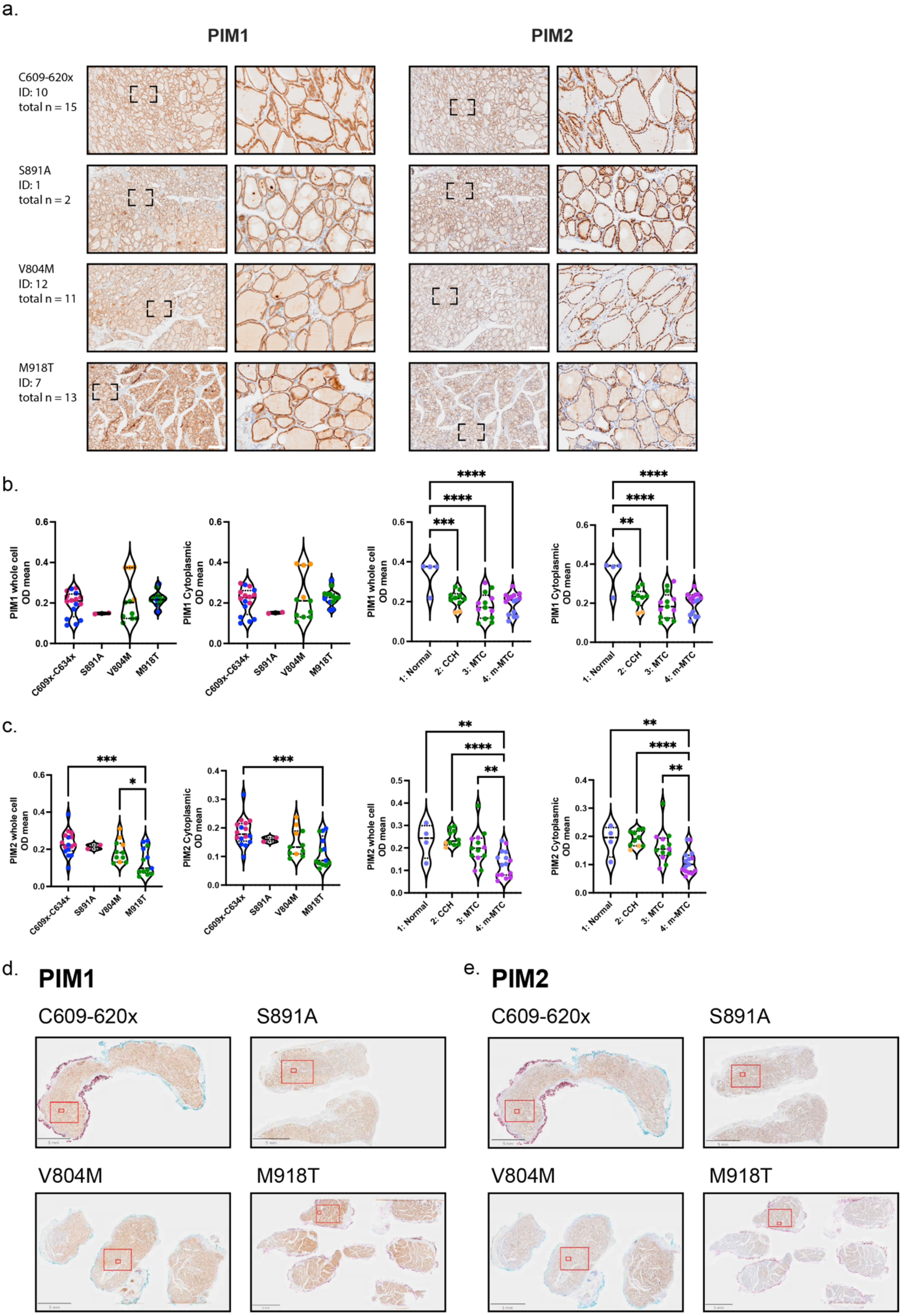
a.
PIM1
PIM2
C609-620x
ID: 10
total n = 15
S891A
ID: 1
total n = 2
V804M
ID: 12
total n = 11
M918T
ID: 7
total n = 13
b.
PIM1 whole cell OD mean
PIM1 Cytoplasmic OD mean
C609x-C634x
S891A
V804M
M918T
1: Normal
2: CCH
3: MTC
4: m-MTC
c.
PIM2 whole cell OD mean
PIM2 Cytoplasmic OD mean
d.
PIM1
C609-620x
S891A
V804M
M918T
e.
PIM2
C609-620x
S891A
V804M
M918T

Supplementary Figure 6. PIM1 and PIM2 protein levels are reduced within metastatic MEN2 thyroid tissues.

a) Representative example of MEN2 thyroid tissues H-DAB stained for either PIM1 or PIM2. For each pathogenic variant example for PIM1 and PIM2, the left image scale bar represents 500 µm, the hashed box shows the location of the image to the right, and the scale bar for each right image show 100 µm. b,c) Quantification of PIM1 and PIM2 whole cell and cytoplasmic protein levels within patient thyroid tissues. The data are grouped by either pathogenic variant or histology type, then coloured by either the histology type or pathogenic variant, respectively (as previously explained in figures 3, 4 and S4). Analysis performed using One-way ANOVA followed by Brown-Forsythe test, * $p < 0.05$, ** $p < 0.01$, *** $p < 0.001$, **** $p < 0.0001$. For tissues stained with PIM1 and PIM2; C609x-C634x $n = 16$, S891A $n = 2$, V804M $n = 11$, M918T $n = 13$: Group 1 (histologically normal) $n = 4$, group 2 (CCH) $n = 10$, group 3 (MTC) $n = 13$, group 4 (m-MTC) $n = 15$ d,e) Representative images of MEN2 patient thyroid tissues. Scale bars show 5 mm.

Supplementary Table 1. Antibodies applied in Histology tumour staining.

Epitope retrieval	Primary Antibody	Catalogue no.	1° dilution	Secondary	Catalogue no.	2° dilution	Detection	Catalogue no.	DAB	Catalogue no.
Citrate buffer pH6	β-catenin	C2206 (Sigma Aldrich)	1:1000	Goat anti-Rabbit biotinylated	BA-1000 (Vector laboratories)	1:250	ABC-HRP kit	PK-6100 (Vector laboratories)	DAB substrate kit	(PK-4100 Vector laboratories)
Citrate buffer pH6	NF-κB p65	8242 (Cell Signalling Technology)	1:800	Goat anti-Rabbit biotinylated						
(AR9961) Citrate buffer pH6	GSK3β	MA5-15109 (Thermo Fisher)	1:50	anti-Rabbit	Staining performed using the Leica Bond Platform (Leica Biosystems)				(DS9800)	
(AR9640) EDTA based pH 9	PIM1	MA5-35347 (Thermo Fisher)	1:300	anti-Rabbit						
(AR9640) EDTA based pH 9	PIM2	MA5-42509 (Thermo Fisher)	1:1000	anti-Rabbit						

MEN2_QuPath_script

2024-10-19

Section 1: Creating a QuPath project directory

a) Create a new folder and label appropriately (e.g. B-catenin).
b) Open QuPath and create a project, when asked select the newly made folder as the project directory (alternatively drop the folder onto QuPath and the software will automatically create a project).
c) Load in the .vsi files of DAB-stained tumours. Select image type. For Beta-catenin and p65 (QuPath v0.3.2), Brightfield H&E was selected for image type. For PIM1, PIM2 and GSK3Beta (QuPath v0.5.1), Brightfield H-DAB was selected.

Section 2: Annotating each tumour

d) Use the Polygon tool to draw around each tumour (it doesn't need to be close to the tumour edge, it's just so the program can identify individual tumours on each slide for the next step).
e) Open QuPath script editor and run the following line of code. This will detect tissue within each annotation based on the pixel classifier. For Beta-catenin and p65 the pixel classifier 'Tissue detector (Random trees Res low Annotations)' was applied. For GSK3[beta], PIM1 and PIM2 the pixel classifier 'Tissue detector (RTrees Res low)3' was applied. New annotations of just tumour tissue regions are created, the original drawn annotations can then be deleted.

```
selectAnnotations();
createAnnotationsFromPixelClassifier("Tissue detector (Random trees Res low
Annotations)", 500000.0, 3000000.0, "DELETE_EXISTING", "SELECT_NEW")
print "Annotations made!"
```

f) Go through each sample to check that all tissue regions have been identified and no non-tissue regions are included in the annotations. Any adjustments to the tissue annotations can be made with the wand tool.

Section 3: Cell detection

g) Perform cell detection using the code below. This detects each cell within the tissue by QuPaths cell segmentation algorithm.

```
selectAnnotations();
// Cell detection
runPlugin('qupath.imagej.detect.cells.WatershedCellDetection',
'{"detectionImageBrightfield": "Hematoxylin OD",  "requestedPixelSizeMicrons"
:
0.5,  "backgroundRadiusMicrons": 8.0,  "medianRadiusMicrons": 0.0,
"sigmaMicrons": 1.2,  "minAreaMicrons": 10.0,  "maxAreaMicrons": 400.0,
"threshold": 0.02,  "maxBackground": 2.0,  "watershedPostProcess": true,
"excludeDAB": false,  "cellExpansionMicrons": 3.0,  "includeNuclei": true,
"smoothBoundaries": true,  "makeMeasurements": true}');
```

Section 4: Data export

h) Once cell detection is completed for all samples in the dataset, the following code exports all cell (detection) data to a .csv file

```
import qupath.lib.gui.tools.MeasurementExporter
import qupath.lib.objects.PathDetectionObject;

// Get the list of all images in the current project
def project = getProject()
def entry = getProjectEntry()
entryList = []
entryList << getProjectEntry()

def outputPath = buildFilePath(PROJECT_BASE_DIR, 'detection results')
mkdirs(outputPath)
def imageData = entry.readImageData()

// Separate each measurement value in the output file with a comma
def separator = ","

// Choose the columns that will be included in the export
// Note: if 'columnsToInclude' is empty, all columns will be included

def columnsToInclude = new String[]{"Image", "Parent", "Cell: DAB OD mean",
"Nucleus: DAB OD mean", "Cytoplasm: DAB OD mean"}

// Choose the type of objects that the export will process
def exportType = PathDetectionObject.class

// Define new file names
def name1 = entry.getImageName() +'_detectionMeasurements.csv'
//need a file here
def outputFile = new File( buildFilePath(outputPath, name1))

def exporter  = new MeasurementExporter()
                  .imageList(entryList)
                  // Images from which measurements will be exported
                  .separator(separator)
                  // Character that separates values
                  .includeOnlyColumns(columnsToInclude)
                  // Columns are case-sensitive
                  .exportType(exportType)
                  // Type of objects to export
                  .exportMeasurements(outputFile)
                  // Start the export process

print "Done"
```

The script described and developed here was based heavily on scripts available at https://qupath.readthedocs.io/en/ as well as others found online.

Supplementary Table 2. Patient cohort summary data.

Information regarding the patient cohort, listed per patient, can be found within the file Supplementary Data 02. Participant number (ID) is the patient ID used throughout this study. *Patient sample excluded from PamGene Kinome array due to loss of sample, **excluded from tumour analyses of both B-catenin and p65 analyses due to varying cell types, ***excluded from tumour B-catenin analyses due to corrupt images. Variant groups state an individual patients variant, Control group nd=not determined. Thyroid status at operation and Histology groupings are designated as follows. Thyroid status at operation; Normal thyroid tissue (n), C-cell hyperplasia (c), MTC (m), MTC with nodal spread (m+n). Histology Group; Normal tissue (1), C-cell hyperplasia (2), Medullary thyroid cancer (3), Metastatic medullary thyroid cancer (4). Number of tumour foci, states the number of thyroid tissue pieces from each patient mounted on slides and analysed independently within this study.
